# Practical Observations on Certain Inflammatory Affections of the Mucous Membranes of the Lungs and Alimentary Canal

**Published:** 1818-10-01

**Authors:** Edward Lyon

**Affiliations:** one of the Physicians to the Manchester Infirmary, &c.


### *Practical Observations on certain inflammatory Affections of the Mucous Membranes of the Lungs and Alimentary Canal.*

By Edward Lyon, M.D. one of the Physicians to the Manchester Infirmary, &c.

The record of the first and third of the following cases was preserved with the view of correcting the errors of my own practice; that of the other two, as a memento of the effects of inflammation on the mucous membrane of the respiratory organs, and of the alimentary canal. If their insertion in your Journal may contribute in any degree to the elucidation of disease, they are very much at your service.

Case I. John Clarke, canal boatman, æt. 22, was admitted into the Manchester Infirmary, on Monday the 16th of February, 1818, affected with considerable dyspnœa, hoarseness, cough, spitting of blood, and pain in the breast: countenance suffused; bowels loose; pulse feeble; tongue pretty clean; thirst. Had been ill a fortnight, after much previous exposure to the weather.

Adhibeantur cucurbitulæ cruentæ pectori, ut mittatur sanguis ad ℥xij. Postea

Imponatur emp. lyttæ pectori.

Capt. * mist. acac. c. scilla f ℥j 3tis horis.

---

* According to a formula used in the Manchester Infirmary.

*Mistura Acaciæ composita.*

℞ Mucilaginis acac. f ℥viij; mist. ammoniaci f ℥ij; aquæ menth. virid. f ℥iv; syrupi f ℥ß. M.

*Mist. Acac. cum Scilla.*

℞ Mis. acac. comp. f ℥xv; Tr. scillæ f ʒjß; — camph. comp. f ℥ß. M.

17th. Not much change. Pulse more full. He was directed to be cupped between the shoulders to the same amount as before, to use a solution of cream of tartar for drink, to continue the mixture, and to take two of the under-mentioned pills* every night.

18th. 11, A. M. Spitting of blood diminished; pain removed; breathing easier; thirst continues; pulse 112.

℞. Mist. acac. comp. f ℥viij; Tæ. digitalis f ʒj. M. sumat f ℥j 4ta quaque hora.

Cont. pil. et potus.

19th. 2 ½, P. M. He is reclining in bed; has been sweating profusely, and is at times very hot; pulse 120, small.

20th. Is up and dressed; appears to catch for his breath every now and then, when he speaks: has a stupid look; says he is purged; pulse 120.

Addatur mist. tæ. digitalis f ʒß.

21st. There has been a considerable flow of blood from the rectum this morning. His cough is not better; sputa no longer bloody; breathing short; countenance of a deep red colour; eyes heavy; says he has no pain.

Omitt. medicamina.

℞. Tæ. opii f ʒß;—catechu f ℥ß; infusi rosæ f ℥vijß. M sumat f ℥j 3tia q. h.

Capt. h. s. † pil. digitalis c. camph. iij.

22d. 1 ½ P. M. Sitting up; general appearance not changed. He has lost no blood to-day; has been breathing hard this morning, and sweating profusely; is easier now. He is said to wheeze a good deal when laid down. Pulse 120.—Cont. med. heri prescripta.

℞. Sp. lavand. comp., sp. ammon. aa f ʒß; mist. camphoræ f ℥j. ft. haustus h. s. sumendus cum pilulis.

23d. 9 ½, A. M. He is lying on his right side; breathing quick, with a slight degree of rattling noise; and his face covered with moisture. The purging has ceased. Pulse 104.—Omitt. med.

---

* Pilulæ hydrarg. submuriat. c. scilla.—℞. Scilla radicis recent. exsiccatæ et contritæ ʒj; zingiberis radicis contritæ ℈ij; hydrarg. submuriat. ʒß; extracti opii gr. xv; confect. rosæ caninæ q. s. sit, M. ft. pil. 60.

† Containing in each pill, of digitalis and opium, each, one-third of a grain; of camphor, one grain.

℞. Hydrarg. submuriat. ℈j; opii gr. iij; confect. rosæ q. s. ft. pil. x: quarum capt. j ter die cum f ℥j mist. sequentis.

℞. Tæ. digitalis f ʒjß; * mist. ammon. acet. ℈ß. M.

24th. Has passed an unquiet night; no amendment; face more deeply suffused; pulse 108, small. Continues the same medicines.

25th. 12, MER. He sat by the fire most of the night, and is now there, breathing hard, with a noise something like that of churning: the bronchiæ seem to be loaded with mucus. Pulse 104. Urine reported to be plentiful. Cough loose.

26th. He sat by the fire last night till half past three o'clock, and then lay down; his respiration became more laborious, and he expired about half past four.

*Inspection of the Body, eleven Hours after Death, by* Mr. MORRIS, *House Apothecary, and other Gentlemen belonging to the Infirmary.*

The lips, eyelids, and nose, are rather livid; the body is yet warm. On laying open the thorax and abdomen, the surface of the lungs appears of a pale colour; and the heart, of somewhat greater bulk than ordinary, is seen lying between them. There are no preternatural adhesions, nor is there any effusion into the thoracic cavity; the quantity of fluid in the pericardium does not exceed an ounce. The lungs and their appendages being removed, and the trachea with some of its branches being slit open, we see the inner membrane of these tubes completely lined with mucus, and exhibiting distinct marks of inflammation; considerably less, however, in the trachea than in its branches. On cutting the substance of the lungs, abundance of frothy mucus issues from every pore. In removing the lungs from their situation, the *vena cava* was divided, and a large quantity of black and fluid blood discharged. The heart now appears of a natural size, and its structure is found to be quite healthy; there is a whitish coagulum adhering to the parietes of the right ventricle, and the mouth of the pulmonary artery is plugged with a clot of black blood.

---

* ℞. Aquæ menth. virid. f ℥vj; liq. ammon. acet. f ℥ij; syrupi f ʒij. M.

The liver is large, but has otherwise a very healthy appearance; the gall-bladder is contracted and empty. The peritoneal coat of the small intestines, of the ileum in particular, exhibits much greater vascularity than is natural. The alimentary canal contains a very small quantity of fæcal matter. The condition of the lungs, and indeed of all the other viscera, previously to this fatal illness, appears to have been remarkably good. I regret that the contents of the cranium were not examined, as it is probable, from the aspect of the patient, and from the ascertained relation between the lungs and the brain, that in this organ there existed some derangement, which contributed to hasten the fatal termination.

A case, in many particulars agreeing with the above, is given by Mr. Bedingfield, in his useful Compendium: the circumstances in which it chiefly differs are, the greater age of the patient, the more acute form of the disease, giving rise to purulent secretion, and the greater rapidity of its course. In the treatment adopted, there does not appear to have been any material difference. That this treatment, in the instance now related, was in all respects the most proper, I am far from being satisfied; and cannot but entertain the hope, that a patient similarly situated might be saved by more vigorous measures. The disease, it is true, had made considerable progress before medical aid was applied for; and this consideration, joined to the feebleness of the pulse, deterred me, perhaps unfortunately, from having recourse to general blood-letting. The abstraction of blood by cupping did afford some relief; but, when we consider that the ultimate effect of this, in lowering the system, is as great as that produced by an equal loss of blood, taken away more suddenly, whilst the immediate impression made by the latter mode is incomparably greater,—it is not unreasonable to think, that previous venesection would have been still more efficacious. The advantage derived from moderate bleedings, in several instances of exasperated chronic catarrhal cough, occurring in elderly people, would induce me, on the future occurrence of a case like the present, to make a pretty full trial of this powerful remedy. The mercurial course, to be (if calculated to be) of any use, should have been commenced earlier. Inhalation of the vapour from hot water might be employed as an adjunct to other remedies.

The following Case, shewing the puriform termination of Bronchitis, occurred in St. Bartholomew's Hospital. I had no opportunity of seeing the patient before his death; and the few particulars, noted below, were communicated by Mr. Wheeler, the respectable Apothecary of that Institution. The dissection was conducted, with his usual accuracy, by Mr. Lawrence.

Case II. Dec. 3, 1811. The body of a man, about 35 years of age, was examined eight hours after death. For the space of two years he had been afflicted with dyspnœa to such a degree, that he could not lie down; his face was usually tumid and purple; he complained of frequent "flustration," as he called it, and a sensation that induced him to lay suddenly hold of any thing that was at hand, in order to save himself from falling.

The first step of the dissection, after removing the integuments, was to lay bare the pleura costalis of the left side, through which the lung was observed in close contact: an aperture was then made in the pleura, the mouth and nostrils being open, and the lung immediately subsided. The same was done on the right side, only that the mouth and nostrils were closed with our fingers; and in this case also the lung subsided, but neither so speedily nor so completely as on the left side: there seemed indeed to be some very slight adhesions at this part.

The abdominal surface of the diaphragm was remarkably flat, and the liver and stomach consequently pushed low into the abdomen. The lungs were uncommonly healthy, with the exception of a slight induration in a small portion of the inferior edge of the right lung.

The heart was observed to be larger than common, particularly the right auricle and ventricle, which were much distended with blood; but there was no derangement of structure.

When the larynx and pharynx were separated from the root of the tongue, and pulled downwards, a purulent matter flowed out of the trachea, to the amount of nearly an ounce. The œsophagus being slit open, was seen to be sound: the larynx was likewise free from inflammation, but the mucus membrane of the trachea, from its commencement downwards to its minutest ramifications, was inflamed, and covered with a purulent matter in considerable quantity; the inflammation was slighter above, and gradually greater lower down; it seemed to be recent, since there was no apparent thickening of the membrane.

The subject of the following case was unfavourably situated for the beneficial operation of medicine, having been reduced from a comfortable and respectable condition to a state of great privation, with a family looking to him for support, which it was out of his power to afford. We are informed in Boswell's Life of Dr. Johnson, that Dr. Butter, of Derby, told Johnson and the author, "that whatever a man's distemper was, Dr. Nichols would not attend him as a physician, if his mind was not at ease; for he believed that no medicine would have any influence. He once attended a man in trade, upon whom none of the medicines he prescribed had any effect. He asked the man's wife privately, whether his affairs were not in a bad way? She said, No. He continued his attendance some time, still without success: at length the man's wife told him, she had discovered that her husband's affairs *were* in a bad way." There cannot, indeed, be a doubt, that mental affections have a very decided influence on disease, acute as well as chronic; and I think myself warranted in tracing a considerable portion of mischief to that source on the present occasion.

Case III. John Unsworth, ætat. 47, naturally of a robust and corpulent habit, was, on Saturday, August 2, 1817, admitted a home-patient of the Manchester Infirmary, labouring under urgent pain of the head and breast, dyspnœa without much cough, and a great degree of thirst, with a foul, dry tongue, and a full, strong, and frequent pulse; several clots of blood have been discharged from the nostrils. He was taken ill ten days ago, after having suffered much from fatigue, cold, and want. On Wednesday last he was freely purged, and experienced some relief from the loss of about eight ounces of blood, which is reported to have been sizy.

The remedies prescribed were, V. S. to 16 or 20 oz. according to the effect; a blistering plaster over the sternum; xv gr. of a powder, containing equal parts of calomel, jalap, and scammony, to be taken in the evening; and an ounce of the following mixture every three hours:

℞. Magnes. sulphat. ℥j; Mist. ammon. acet. f ℥xj; Liq. antimon. tart. f ʒvj. M.

3d. The blood taken amounts to about 20 oz.; a portion of about 4 oz. in the second cup is covered with a thin coat of buff, exhibiting fibrous streaks and a greenish tinge;* that which was first drawn is not so. He has been

* It is observed by Dr. Pemberton, when laying down the treatment of hepatitis, in his work on Diseases of the Abdominal Viscera, that, if the much disturbed all night by diarrhœa, which began at six o'clock last night, before the powder was taken. He was rather delirious; the blister has risen well. Respiration seems easier, and he complains less of his head. Intermitt. mist. aperiens.

℞. Oxymel. scillæ f ℥ß; liq. antimon. tart. f ℥iß; tincturæ opii ♏ xx; aquæ menth. virid. f ℥x. M.—Capt. f ℥j. 3tia quaque hora.

℞. Hydrarg. submuriat. gr. v; opii gr. j; confect. rosæ, q. s. ft. pil. ij. h. s. sumendæ.

4th. He passed another bad night, in vehement struggles for breath; is easier now. Pulse full, and rather hard; tongue coated with a yellowish white fur. Was ordered to resume the aperient mixture.

Mr. Barker (physician's clerk) saw him last night, and took eight ounces of blood from his arm; ordered a cathartic powder and an anodyne mercurial pill, with the saline aperient mixture and a linctus for the cough. He passed a comfortable night, and is considerably better now. Tongue cleaner at the edges; pulse reduced.

6th. Yesterday evening, being worse, he was again bled with great advantage. Continues his medicines.

7th. An unsuccessful attempt to bleed him again was made last night. He breathes better, and does not complain of pain; but he is rather wandering in his talk, and agitated, rising up in bed with hurry on my approach. A blister was applied, *inter scapulas,* early this morning.

8th. He continues much the same, with respect to cough and respiration, and manifests equal agitation and incoherence. Feels a desire to go to stool, but can effect nothing; complains of his legs and feet being cold; pulse more frequent. Ten leeches were directed to be applied on his temples, and a powder containing five grains of calomel, and fifteen of rhubarb, to be taken immediately.

11th. On the 9th, after ineffectually taking some purgatives, a glyster was administered, which brought away a large quantity of green fæces. He has been very restless and delirious.

Capt. Infusi digitalis f ℥j. cum spt. ætheris nit. f ʒß 4ta quaque hora.

---

buff is flat, semi-transparent, of a greenish yellow colour, and with a striated filamentous texture running through it, we must refrain from any furthergeneral bleeding.

12th. Has experienced some violent retching this morning, which he had yesterday also in a less degree. Is capable of answering questions, if roused; but is frequently getting out of bed without apparent object. Has made a large quantity of clear, dark-coloured urine. Tongue white, as formerly, tolerably moist at the edges. Had a green stool yesterday afternoon, and another at three this morning.

Ten more leeches were ordered to be applied to the temples; a blistering plaster behind each ear; the infusion to be continued, and two cathartic pills to be taken night and morning.

13th, 10 A. M. He is more *insensive;* has been very restless; is now quiet; eyes fixed; frequently mutters in a hurried, yet cautious tone, "Take care!" Seems to have some tenderness in the epigastre, putting away my hand when I press there; feet cold; pulse failing. No evacuation from the bowels since yesterday morning; the pills having been spit out of his mouth. He expired about one o'clock.

14th. 4½ P. M. The body was inspected by Messrs. Bingham and Barker and myself. The following is a brief account of what we observed.

*Head.* The dura mater adhered strongly to the skull-cap, and there seemed to be an unusual redness of that membrane in the part corresponding to the vertex. There was a degree of milkiness over the pia mater, but nothing very striking; a little water in the ventricles.

*Thorax.* Firm adhesions of the pleura on each side, with inflammatory redness of the pleura investing the inferior lobe of the right lung; lungs not unhealthy.

*Abdomen.* Effusion of serum within the peritoneum to the amount of about two pints. Liver tuberculated, exhibiting, when cut into, small orange-coloured molecules; inner coat of the stomach inflamed, the prominent rugæ presenting a dusky red colour, owing to distended blood-vessels. Intestines distended with air; spleen of a pale blue colour, speckled with white; kidnies of a dusky red, and large.

I have but few additional comments to make on this case. With regard to the infusion of digitalis, however, it would scarcely have been ordered, had inflammation of the stomach been suspected; and, indeed, in acute diseases generally, my trials of this medicine do not offer many temptations to extend its employment; though, in chronic affections, it often accomplishes all that can reasonably be expected from medicine. The obscure indications of the presence of gastritis, which was little suspected until a short time before death, may be worthy of notice, as it is by no means uncommon to find traces of this, or a similar affection, on a post obit inspection of bodies in which it had not previously been supposed to exist. It is probable, that no considerable deviation from the natural colour and consistence of the fæces takes place, without more or less of an inflammatory action in the mucous membrane of some part of the alimentary canal. The subsequent Case occurred in St. Bartholomew's Hospital: it exhibits a complication of disease.

Case IV. December 21, 1811. Eight hours after death, the body of a man was examined who had been in the hospital a fortnight; the previous duration and circumstances of his complaint were not known, except that he had no evacuation *per anum* for twelve days before his admission.

The symptoms observed in him were, a continual vomiting of all he swallowed, and of a greenish matter; an aversion to speaking, though he did not complain of pain when pressure was made upon the abdomen; costiveness, and an intermitting pulse.

In the stomach was found a large quantity of matter like that which he vomited; and the contents of the small intestine were of the same nature. The mucous coat of the stomach was of a dusky red colour, very different from that which usually attends active inflammation; and this colour was more intense on the prominent part of the rugæ than elsewhere. The same appearance was observed, more or less, along the whole line of small intestine, and on a few inches of the ascending colon. A firm clot of blood was found adhering to the inner coat of the jejunum; a clot was also found on each psoas muscle, in the course of the spermatic vessels. The fæces in the colon were of a very bad kind, soft, dark coloured, and fœtid; yet the liver appeared remarkably healthy, and the gall-bladder contained a good portion of perfect bile.

The pericardium adhered universally to the heart by flakes of coagulated lymph, which seemed to have been recently effused; the heart itself was sound. The lungs were in some parts indurated, and these parts sank in water. More water than is natural was found in the ventricles of the brain.

It was remarked that the blood, in all parts of the body, contained a large portion of air; and that the quantity of blood was small in proportion to the size of its containing vessels.

EDWARD LYON, M. D.

Manchester, July, 1818.

---

